# Application of CRISPR/Cas Genomic Editing Tools for HIV Therapy: Toward Precise Modifications and Multilevel Protection

**DOI:** 10.3389/fcimb.2022.880030

**Published:** 2022-05-25

**Authors:** Alexandra Maslennikova, Dmitriy Mazurov

**Affiliations:** ^1^Cell and Gene Technology Group, Institute of Gene Biology of Russian Academy of Science, Moscow, Russia; ^2^Center for Precision Genome Editing and Genetic Technologies for Biomedicine, Institute of Gene Biology of Russian Academy of Science, Moscow, Russia

**Keywords:** HIV, CRISPR/Cas, knock-in, C-peptides, co-receptors, restriction factors, CD4 T cells, B cells

## Abstract

Although highly active antiretroviral therapy (HAART) can robustly control human immunodeficiency virus (HIV) infection, the existence of latent HIV in a form of proviral DNA integrated into the host genome makes the virus insensitive to HAART. This requires patients to adhere to HAART for a lifetime, often leading to drug toxicity or viral resistance to therapy. Current genome-editing technologies offer different strategies to reduce the latent HIV reservoir in the body. In this review, we systematize the research on CRISPR/Cas-based anti-HIV therapeutic methods, discuss problems related to viral escape and gene editing, and try to focus on the technologies that effectively and precisely introduce genetic modifications and confer strong resistance to HIV infection. Particularly, knock-in (KI) approaches, such as mature B cells engineered to produce broadly neutralizing antibodies, T cells expressing fusion inhibitory peptides in the context of inactivated viral coreceptors, or provirus excision using base editors, look very promising. Current and future advancements in the precision of CRISPR/Cas editing and its delivery will help extend its applicability to clinical HIV therapy.

## Introduction

HAART has decreased the incidence of acquired immunodeficiency syndrome (AIDS) and mortality among HIV-infected patients by suppressing HIV replication. It is impossible to eradicate HIV with HAART because it does not affect the HIV reservoir, which represents the transcriptionally inactive proviral DNAs integrated into the host genomes of the long-lived circulating human memory T cells. Upon the cessation of HAART, the latent HIV activates and starts to spread by infecting new target cells—mainly CD4 lymphocytes—which ultimately leads to disease progression. To avoid this, HIV-infected individuals must regularly and for their entire lives, take antivirals. However, long-term anti-HIV therapy can be toxic; furthermore, the virus may become resistant to the drugs (Liu et al., 2014; Sluis-Cremer, 2014) that requires modification of the HAART regimen.

**Figure 1:**
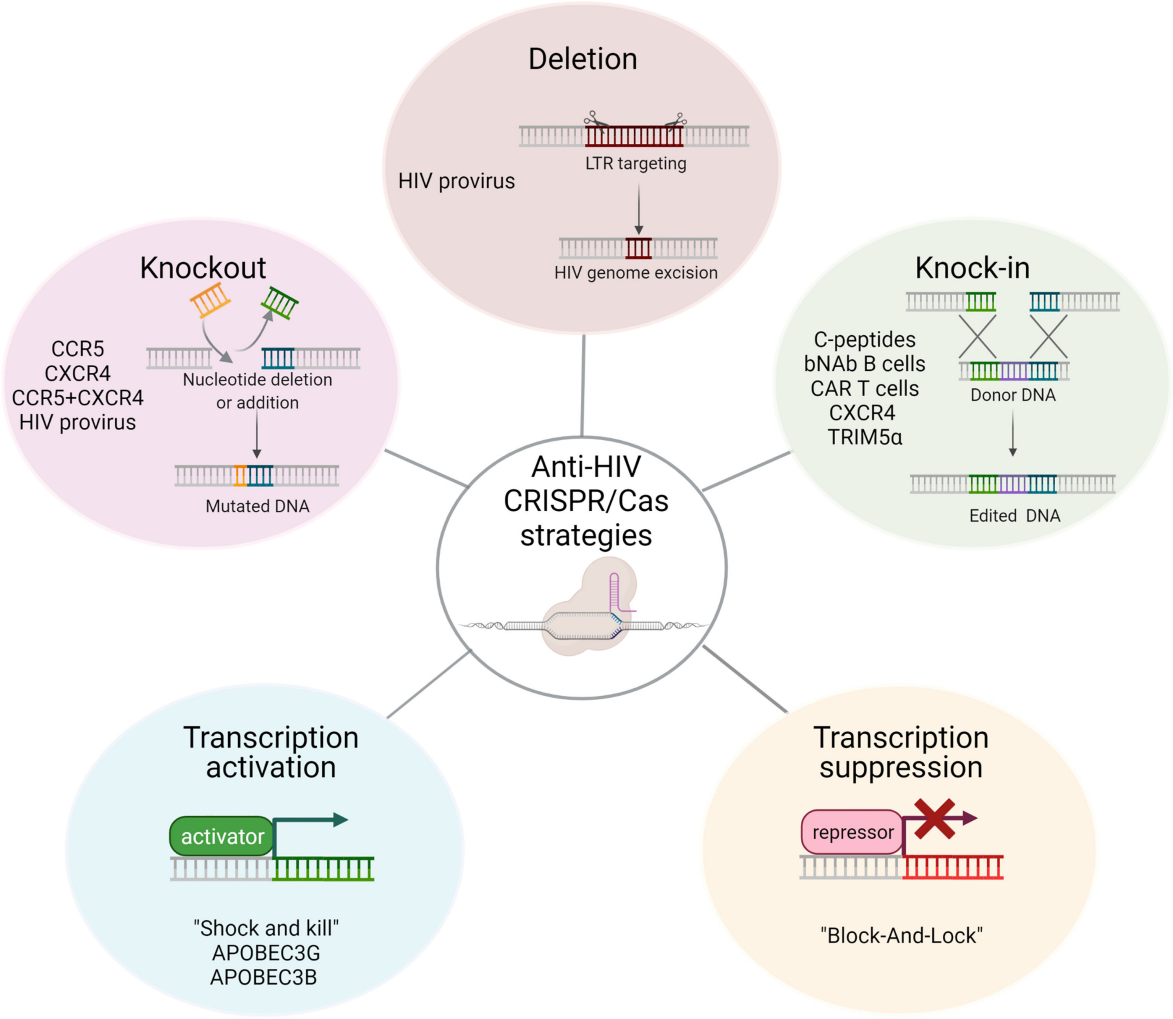
A flowchart showing CRISPR/Cas anti-HIV approaches used to protect cells from HIV infection. Depending on the gene-editing techniques (shown on top of each block) and cellular or viral genes selected for targeting (shown below) they were divided into five groups. Some strategies may overlap. For example, gene knockout or provirus deletion can be accompanied by targeted integration of a therapeutic gene; all of them are inheritable and effects are stable in comparison to CRISPR-based transcription regulation. This image was created with BioRender.

Programmed genomic nucleases are a promising technology for combating HIV infection, primarily the clustered regularly interspaced short palindromic repeats (CRISPR) and CRISPR-associated endonuclease 9 (Cas9). CRISPR/Cas represents a system of adaptive immunity in bacteria and archaea against viral and plasmid invasion. It has been engineered to introduce targeted gene modifications into the genomes of different eukaryotic cells. Cas9 nuclease has become the most popular genome editor with a broad range of applications. Depending on the Cas9 variant used, the type of DNA reparation, and whether viral or cellular genes were targeted, we divided anti-HIV CRISPR/Cas strategies into the following categories (**Figure 1**): knock-out (KO) of viral coreceptors CCR5 and CXCR4; excision or inactivation of proviral DNA; knock-in of therapeutic genes, peptides, or introducing precise mutations in viral restriction factors and entry molecules; transcriptional activation of viral and cellular promoters for the “shock and kill” therapy or activation of the silent restriction factors, respectively; virus repression or “block and lock” strategy. Despite the differences in targeting mechanisms, all of these strategies are intended to expand the HIV-resistant population of CD4 lymphocytes and shrink the reservoir of latent HIV to ultimately lead to a “functional cure,” a balanced condition in which the virus does not replicate at minimal doses of HAART or absent treatment. Each of the anti-HIV CRISPR/Cas strategies discussed below has some advantages and disadvantages in terms of efficiency, safety and applicability, which we summarized in **Table 1**.

**Table 1:** Pros and cons of different anti-HIV CRISPR/Cas strategies.

Effect on DNA	Viral/host target	Editing system	Pros	Cons	Citations
1. Knockout	CCR5	Cas9, Cas12a	Applicable for HSPC	Difficult KO selection due to low expression, no effect on X4-tropic virus	(Mandal et al., 2014; Kang et al., 2015; Hultquist et al., 2016; Xu et al., 2019; Liu et al., 2020; Lin et al., 2021)
	CXCR4		Easy KO selection	Not suitable for HSPC and against R5-tropic virus	(Hou et al., 2015; Schumann et al., 2015)
	CCR5+CXCR4		Tropism independency	Reduced efficiency of biallelic KO of both coreceptors	(Yu et al., 2017)
	HIV provirus mutation		High efficiency	Viral escape mutant emergence, not all HIV proteins are inactivated	(Ueda et al., 2016; Wang et al., 2016b; Yoder and Bundschuh, 2016)
	HIV provirus deletion		True eradication	Reduced efficiency, more off-targets, chromosomal rearrangements	(Hu et al., 2014; Dash et al., 2019; Campbell et al., 2019; Gao et al., 2020)
2. Knock-in	TRIM5α (R332G/R335G)	Cas9	Responsiveness to IFNγ stimulus, no escape mutants	Requires biallelic KI for full activity, moderate inhibition of HIV replication	(Dufour et al., 2018; Désaulniers et al., 2020)
	C-peptides at CXCR4 locus		Strong and wide resistance to HIV, possibility of coreceptor KO	Low HDR efficiency, only edited cells are resistant to HIV, a possibility of viral resistance emergence	(Maslennikova et al., 2022)
	bNAb at IgH locus of B cells		Protection of non-edited cells, effective at low HDR	Possible escape mutant generation and less effective against virus cell-to-cell transmission	(Hartweger et al., 2019; Nahmad et al., 2020)
	bNAb at CCR5 locus of T cells	megaTAL	Protection of effector CAR T cells from HIV infection	Low HDR efficiency, resistance of latently infected lymphocytes to killing	(Hale et al., 2017)
3. Gene activation	HIV provirus, «shock & kill»	dCas9-VP64	Shrinks reservoir of HIV latency, high specificity of activation, SAM is the most powerful activator	Does not eradicate HIV, transient nature of activation, non-responsiveness of some latent proviruses	(Saayman et al., 2016)
		dCas9-SAM			(Zhang et al., 2015; Bialek et al., 2016)
		dCas9-SanTag			(Ji et al., 2016; Bialek et al., 2016)
		dCas9-p300	Prolonged activation		(Limsirichai et al., 2016)
	APOBEC3B	dCas9-SAM	No genetic modifications, boosting natural resistance	Potential involvement in cancer development, moderate antiviral effect	(Bogerd et al., 2015)
	BST-2			Enhances cell-to-cell transmission of HIV	(Zhang et al., 2019)
4. Gene repression	HIV provirus, «block & lock»	dCas9-KRAB	Prolonged suppression without genotoxic effects	Does not eliminate HIV reservoir in organism	(Olson et al., 2020)

## Effector CAS Nucleases as Genome-Editing Tools

### Type II Cas9 Nuclease

In 2012, Emmanuelle Charpentier and Jennifer Doudna demonstrated for the first time that the RNA-guided CRISPR-associated DNA nuclease Cas9 can be programmed to edit the genomes of eukaryotes (Jinek et al., 2012). In 2020, they received the Nobel Prize in Chemistry for the research on CRISPR/Cas system. The bacterial CRISPR/Cas system is divided into two classes and six types (Mosterd et al., 2021). The effector complex of the class I CRISPR system consists of several Cas subunits, whereas the class II system is represented by a single effector nuclease protein. The simplicity of expressing a single Cas protein has led to the widespread adoption of the class II CRISPR/Cas system for genome editing in humans and other organisms. In bacteria, CRISPR/Cas functions to acquire spacer sequences from viral DNA, integrate them into the bacterial CRISPR cassette, and then use those sequences to protect the bacteria from invasion by the same virus. The three phases of this process are called adaptation, expression, and interference. However, only the last phase of this process is relevant to the development of new genomic editors.

The most popular effector nuclease is Cas9 from *Streptococcus pyogenes*. It is a class II type II CRISPR/Cas and requires trans-activating RNA (tracrRNA) and crRNA to bind with a target DNA. To simplify Cas9 DNA targeting, the tracrRNA and crRNA have been fused to a single guide RNA (sgRNA) and expressed under the U6 promoter. The sgRNA-Cas9 complex has been shown to efficiently and specifically target different genes in different types of human cells (Cong et al., 2013; Mali et al., 2013). The Cas9 crRNA consists of the twenty-nucleotide spacer sequence that recognizes the target strain of dsDNA flanked by the NGG 3’-protospacer adjacent motif (PAM). In this review, we will briefly mention the major advances in Cas9-based editing tool development, as more details can be found in other, specific reviews. The most significant Cas9 modifications and applications are listed in chronological order: double-nicking with Cas9n to reduce off-targeting (Ran et al., 2013), the GeCKO knockout library (Shalem et al., 2014), the synergistic activation mediator (SAM) for gene activation (Konermann et al., 2015) and the Cas9-KRAB repressor (Gilbert et al., 2013), high-fidelity Hypa and Sniper Cas9 (Lee et al., 2018), the prime editor (Anzalone et al., 2019), and the combined nucleotide base editor SPACE (Grunewald et al., 2020).

### Type V Cas Nucleases

Unlike type II nucleases, type V CRISPR/Cas effector proteins contain a single catalytic RuvC domain that cleaves each strand of dsDNA sequentially. Most of the type V nucleases exhibit bystander ssDNA cleavage activity that is used to develop DNA diagnostic tests. The first type V nuclease described was Cas12a or Cpf1. Compared with Cas9, Cas12a is slightly smaller and does not require tracrRNA to bind the crRNA, so the guide RNA is about half as long as that of Cas9. PAM (predominantly TTTV) is located at the 5’-end of the crRNA-binding DNA and is T-rich, unlike the Cas9 G-rich 3’- PAM. Cas12a leaves sticky ends with 4–5 nucleotides, while Cas9 cuts to form blunt ends (Safari et al., 2019). After several mutations were introduced into the *Acidaminococcus* Cas12a sequence, it achieved equivalent on-target activity as Cas9 (Kleinstiver et al., 2019; Zhang et al., 2021) and, since that time, Cas12a has become a second powerful genome-editing tool alongside Cas9. The main advantage of Cas12a, as well as other type V nucleases, is that it can bind to a crRNA cassette directly and release a crRNA from the CRISPR array. This process is very efficient and this feature was used to construct a single vector to express both Cas12a and crRNAs under the control of a Pol II promoter. This feature facilitates multiplex gene editing and regulation of CRISPR/Cas12a (Campa et al., 2019).

Recently, two miniature nucleases, Cas12j (CasΦ) (Pausch et al., 2020) and Cas12f (Cas14) (Harrington et al., 2018) with about half the size of Cas12a have been discovered. Interestingly, Cas12j was isolated not from bacteria or archaea but from huge phages that may adapt it to compete with other phages (Pausch et al., 2020). The miniature Cas proteins would be highly desirable when size is a critical parameter, such as for Cas delivery with an AAV vector. These small Cas proteins are still poorly adapted for genome editing in mammalians. However, recent efforts aimed at improving Cas12f editing efficiency *via* extensive guide RNA modifications (Kim et al., 2022) suggest that the Cas12 subfamily of nucleases will become powerful instruments for human genome engineering.

## Gene Knockout Strategies in HIV Therapy

Catalytically-active Cas9 cuts both strands of dsDNA, producing blunt ends and thereby activating cellular DNA repair mechanisms. In the absence of a donor template, DNA ends are repaired with the nonhomologous end-joining (NHEJ) mechanism. NHEJ is a highly error-prone process typically leading to the addition or removal of a few nucleotides (indels) at a DNA break site. In a coding region, this shifts the open reading frame (ORF) and results in gene knockout (KO). Because the NHEJ pathway of DNA repair is dominant in all types of eukaryotic cells, intentional gene KO is easily achievable in primary human cells if the CRISPR/Cas is delivered efficiently. In anti-HIV strategies, ORF inactivation has been applied to KO viral chemokine coreceptors or to mutate or excise proviral DNA.

### CCR5 Knockout

HIV uses the cell receptor CD4 (Dalgleish et al., 1984; Klatzmann et al., 1984) and the CXCR4 or CCR5 chemokine receptors to enter the cell (Deng et al., 1996; Feng et al., 1996). HIV’s ability to bind to a specific chemokine receptor is called tropism. Strains of HIV that bind to CCR5 are called R5-tropic; those that bind to CXCR4 are X4-tropic. There are dual tropic strains of HIV that can use either CCR5 or CXCR4. R5-tropic strains are the most frequently transmitted and predominate in the early stages of infection, while X4-tropic strains appear as the disease progresses (Connor et al., 1997; Scarlatti et al., 1997; Weiss, 2013).

A natural mutation in the CCR5 gene, CCR5-Δ32, in a homozygous variant provides resistance to HIV infection due to the lack of CCR5 expression on the cell surface (Samson et al., 1996). The first instance of HIV being cured was reported for the Berlin patient Timothy Ray Brown, who received two transplantations of CCR5-Δ32/Δ32 allogeneic bone marrow and stem cells as a treatment for acute myeloblastic leukemia in 2007–2008. After HAART cessation and until his death from leukemia in 2020, no HIV resurgence was detected (Hütter et al., 2009; Allers et al., 2011; Yukl et al., 2013). This case inspired researchers to develop genetic tools for CCR5 KO in autological patients’ immune cells in an attempt to circumvent the problem of a very limited number of CCR5 -/- donors suitable for allogeneic bone marrow transplants. Before the CRISPR era, Sangamo Bioscience had developed a CCR5-specific zinc finger nuclease (ZFN) and initiated the first HIV clinical trials in 2008. This study demonstrated a moderate improvement in CD4 lymphocyte counts and viral load and requirement for repeated exfusions, *ex vivo* CD4 lymphocyte treatments and reinfusions for a sustained decrease in viral loads (Tebas et al., 2014). Recently, the second case of cured HIV was reported for the London patient who, after analytical treatment interruption, demonstrated HIV remission for more than 30 months (Gupta et al., 2019; Gupta et al., 2020).

With the adaptation of the CRISPR/Cas9 system to human genes, multiple groups reported robust and efficient inactivation of the CCR5 gene, which conferred strong resistance to R5-tropic HIV in edited cells. This was demonstrated using peripheral blood CD4 lymphocytes (Li et al., 2015; Hultquist et al., 2016), induced pluripotent stem cells (iPSC) (Ye et al., 2014; Kang et al., 2015; Lin et al., 2021), and CD34^+^ hematopoietic stem and progenitor cells (HSPC) (Mandal et al., 2014). Engineered CCR5-negative lymphocytes can survive and expand during HIV replication, as shown in *in vitro* cell culture (Wang et al., 2014), humanized mice (Holt et al., 2010), and human patients (Tebas et al., 2014; Xu et al., 2019), suggesting that edited cells receive an additional selective pressure *in vivo* when homeostatic regulation seeks to restore the CD4 lymphocytes lost during viral infection. This presents an obvious advantage for therapy in humans. Autologous iPCSs or HSPCs are very attractive target cells for gene therapy as they can avert the problems related to graft-to-recipient compatibility or the regeneration of terminally differentiated cells. However, compared with CD4 lymphocytes, their usage raises more concerns regarding potential CRISPR/Cas9-mediated off-target mutations that will be inherited by the lineage-committed and terminally differentiated cells produced from progenitor and stem cells. These types of cells are more difficult targets for CRISPR editing than differentiated lymphocytes (Modarai et al., 2018). The substantial enhancement of CCR5 biallelic KO efficiency in iPCSs (Kang et al., 2015; Lin et al., 2021) and HSPGs (Mandal et al., 2014) has been achieved with two sgRNAs. While all of these studies and others (Ye et al., 2014) demonstrated minimal off-target effects and that iPCSs and HSPCs could properly differentiate, the risk of unwanted mutations increases with the number of gRNA targets used per genome, raising more concerns for using this method in clinical treatment.

In 2019, Lei Xu et al. reported successfully transplanted allogeneic CCR5-ablated HSPCs with a survival rate of up to 19 months in a Chinese patient with HIV-1 infection and acute lymphoblastic leukemia (Xu et al., 2019). However, due to the low KO efficiency (5–8%), the analytical HAART interruption resulted in a quick viral rebound. At the end of 2018, Dr. He Jiankui from the Southern University of Science and Technology (Shenzhen, China) reported, at the Second International Summit on Human Genome Editing in Hong Kong, the birth of twin girls, Lula and Nana, who carried loss-of-function CRISPR/Cas9-induced mutations in the *CCR5* gene. This case was widely condemned by the international scientific community (including scientists from China) based on ethical and technical concerns (Davies, 2019).

In summary, CCR5 KO is one of the most promising treatment approaches for HIV because it powerfully blocks R5-tropic HIV entry to cells, does not harm lymphocyte function, and can be achieved at a higher magnitude of efficiency than any knock-in-based editing procedures. Meanwhile, CCR5 on primary T lymphocytes is characterized by a low level of expression and is detectable only in a portion of activated cells, complicating the discrimination of CCR5-null cells from incomplete KOs or unedited cells. Hence, *ex vivo* edited lymphocytes are not usually separated before reinfusion, which decreases the procedure’s therapeutic potential.

### CXCR4 Knockout and Double Coreceptor Knockout

Because HIV can utilize two chemokine coreceptors for entry, CCR5 KO therapy of many patients who had adhered to HAART for a long time may not be effective due to the dominance of X4-tropic strains of HIV. Furthermore, Kordelas et al. reported a shift of HIV tropism from R5 to X4 in the patient who received stem-cell transplantation from a homozygous CCR5 Δ32 donor, indicating that viral escape mechanisms can derail the CCR5 KO strategy (Kordelas et al., 2014). Hence, a CXCR4 or dual coreceptor KO approach would be advantageous to generate tropism-independent resistance to HIV. However, the CXCR4 receptor plays a key role in the development of human hematopoietic/progenitor cells and the differentiation of thymocytes (Hernandez-Lopez et al., 2002; Dar et al., 2006; Nie et al., 2008). This limits the choice of cell types suitable for therapeutic CXCR4 KO. However, experiments on mice with T cell-specific conditional CXCR4 KO (embryonic KO is lethal) demonstrated that mature post-thymic CD4 lymphocytes retained their functionality after KO (Chung et al., 2010). Thus, disrupting the *CXCR4* gene in human peripheral CD4 lymphocytes can be provisionally considered a therapeutically relevant anti-HIV strategy. As in CCR5, genetic ablation of CXCR4 through either ZFN (Yuan et al., 2012) or CRISPR/Cas9 delivered *via* lentiviral vector (LV) transduction (Hou et al., 2015) or ribonucleoprotein complex (RNP) electroporation (Schumann et al., 2015) has proven robust and confers a strong resistance of engineered cells to X4-tropic strains of HIV-1, which provides a growth advantage for them over permissive lymphocytes *in vitro* and *in vivo.*

The most reliable way to generate resistance to dual-tropic HIV is to knock out both viral coreceptors. This requires at least two sgRNAs targeting two genes. The possibility of simultaneously inactivating the *CCR5* and *CXCR4* genes with CRISPR/Cas9 in cell lines and primary CD4+ lymphocytes has been demonstrated by delivering CRISPR/Cas9 using a single LV vector (Liu et al., 2017) or RNP nucleofection (Yu et al., 2017). Of 55% CCR5^-^ and 35% CXCR4^-^ GHOST cells, only 9% carried four bi-allelic gene disruptions; these values were 3-to-5-fold lower in primary edited lymphocytes (Yu et al., 2017).

In summary, CXCR4 gene ablation can be applied to differentiated human lymphocytes either individually or in combination with CCR5 disruption. Combined KO will provide the strongest and broadest resistance of engineered cells to HIV infection, albeit at the cost of significantly lower editing efficiency and likely higher off-target mutagenesis.

### HIV Provirus Inactivation and Deletion

Integrated into the host genome, proviral DNA represents a latent reservoir of HIV infection (Blankson et al., 2002; Siliciano et al., 2003) that can be eradicated only through DNA surgery techniques. Unlike cellular genes, the HIV genome is characterized by a high level of diversity and the expression of multiple RNA splice forms; therefore, the sequences that are most conserved and most important for viral replication, such as LTR or structural and enzymatic gene ORFs, especially those overlapping with frame-shift signals, splice signals, or *cis* elements, should be considered for CRISPR/Cas9 targeting. Many early studies showed that CRISPR/Cas9 can effectively inactivate HIV-1 proviruses by targeting different regions of the viral genome (Ebina et al., 2013; Hu et al., 2014; Liao et al., 2015; Zhu et al., 2015), leading to much excitement in the field. However, shortly afterward, it became clear that NHEJ-mediated indels at the sgRNA target sites could generate viable HIV mutants that were resistant to re-cleavage by Cas9 and responsible for viral rebound later in the infection (Ueda et al., 2016; Wang et al., 2016b; Wang et al., 2016c; Yoder and Bundschuh, 2016). In attempts to control escape mutant generation, multiplexing several sgRNAs targeting the same viral gene (Wang et al., 2018; Ophinni et al., 2020; Vergara-Mendoza et al., 2020) and the deletion of differently-sized proviral segments using one gRNA specific to LTR and one directed to *gag* (Kaminski et al., 2016; Wang et al., 2016a; Yin et al., 2016; Yin et al., 2017; Wang et al., 2018), *pol* (Yin et al., 2016; Yin et al., 2017), *tat/rev* (Liao et al., 2015; Wang et al., 2016a) or *gag* and *pol* simultaneously (Yin et al., 2017; Wang et al., 2018) have been successfully tested. After NHEJ-mediated mutations that inhibit HIV replication, the infected cell may continue to produce certain viral proteins (Kaminski et al., 2016; Yin et al., 2017) which will continue to exert pathogenic effects and deregulate immune responses (Barillari and Ensoli, 2002; Witkowski and Verhasselt, 2013; Langer et al., 2019; Isaguliants et al., 2021). To address this, larger genetic deletions and, ideally, those covering the entire viral genome, have an advantage over local mutations for HIV eradication. However, the design of CRISPR/Cas9 genetic deletions utilizing a pair of sgRNAs and NHEJ reparation mechanism entails two caveats. First, the frequency of deletion events is reported to be markedly lower than that of mutations and decreases with increasing size of the deleted fragment (Canver et al., 2014), i.e., instead of deletions, many HIV genomes will have two mutations; and second, as outlined above, increasing the number of sgRNAs increases the likelihood of unwanted mutations and different chromosomal rearrangements (Maddalo et al., 2014). Along with the dominant strain of HIV present in a patient, many viral quasi-species have been found as well; the later ones may have mismatches at protospacer (sgRNA-targeted) sequences that will facilitate their selection and create resistance to CRISPR/Cas9 therapy (Dampier et al., 2014; Roychoudhury et al., 2018).

Perhaps the most advanced approach for HIV eradication is the entire deletion of proviral DNA with a single sgRNA, which targets identical sequences in the 5’-LTR and 3’-LTR (Hu et al., 2014). The application of one sgRNA not only minimizes off-targeting but may result in more synchronized cleavage of DNA at two sites, thereby increasing the chance of provirus deletion. However, due to sequence variability in this region and the large size of the removable provirus, additional sgRNAs designed to delete large regulatory and exonic pieces of HIV DNA have been preferred in recent studies for their highly efficient latent virus excision *in vitro* (Campbell et al., 2019) and *in vivo* (Yin et al., 2017; Bella et al., 2018), and even complete elimination in model humanized mice when combined with long-acting slow and effective release antiviral therapy (LASER ART) (Dash et al., 2019). Interestingly, Zongliang Gao et al. reported the complete inactivation of the HIV provirus using Cas12a and a single crRNA, which they connected to the specific cleavage pattern of Cas12 outside the PAM-seed region and the predominant formation of deletions at a DNA target site (Gao et al., 2020). Because HIV sequences are rich in A-T nucleotides, editing with Cas12a, which recognizes T-rich PAM, presents more opportunities for target sequence selection compared with Cas9, which improves the likelihood of its applicability for HIV eradication.

In conclusion, the entire elimination of HIV proviral DNA *via* CRISPR/Cas surgery is preferable for HIV eradication compared with local inactivation. In 2021, the Food and Drug Administration (FDA) approved the first clinical trial of the EBT-101 preparation representing AAV-packed CRISPR/Cas9 with two gRNAs targeting three sites in the HIV genome (NCT05144386 at ClinicalTrials.gov). The capacity of EBT-101 to eradicate HIV by deleting most of the viral DNA had been proven in preclinical studies, as Excision BioTherapeutics announced in a press release. However, when considering this strategy for clinical use, it should be remembered that the percentage of latently infected cells in individuals will be much lower than the up to 100% latent infection modeled in cell cultures or animals and will reduce the efficacy of the eradication procedure.

## HDR-Based Therapies for HIV

The precise correction of mutations or targeted delivery of therapeutic genes is a longstanding goal in the field of gene-editing technologies. This can be achieved with CRISPR/Cas in the presence of a repair DNA template when the cellular machinery replaces a dsDNA break with a sequence located between the homology arms of the donor, the process called homology-directed repair (HDR). Because HDR is active in the S/G2 phase of the cell cycle, many primary human cells, especially quiescent ones, are resistant to precise genome modification, making this technology difficult to translate to clinical practice. However, the recent progress in HDR-based gene editing is encouraging. Below, we will discuss the recent advancements in this field, including our own studies, concerning HIV infection.

### Oligonucleotide Knock-In for Precise CXCR4 and TRIM5α Mutation

Single-stranded oligodeoxynucleotides (ssODNs) offer an ideal repair template for small modifications with minimal risk of HDR-independent insertional mutagenesis. The study from Alexander Marson’s lab has reported an efficient CXCR4 KO after nucleofection of Cas9 RNPs with an ssODN repair template that introduced a frame-shifting restriction site; the majority of primary CD4^+^ T cells expressed a CXCR4 null phenotype and one-third of these cells carried the KI genotype (Schumann et al., 2015). Thus, ssODN KI can complement NHEJ-mediated coreceptor KO. Liu et al. used a two-step procedure comprised of HDR-based integration of the selective *puroΔtk* gene followed by its seamless removal with the *piggyBac* transposon to introduce a P191A mutation into the *CXCR4* gene (Liu et al., 2018), which has previously been shown to confer resistance to HIV without significantly affecting coreceptor function (Tian et al., 2005).

Restriction factors represent another option to combat HIV. These are cellular proteins counteracting HIV replication at different stages of the viral life cycle through different mechanisms that, in turn, can be antagonized by viral accessory or structural proteins (Chemudupati et al., 2019; Zotova et al., 2019c). TRIM5α is an interferon-inducible intracellular E3 ubiquitin ligase that acts mainly by binding to and sequestering retroviruses’ capsid proteins in the cytoplasm, blocking virus uncoating (Stremlau et al., 2004). This activity is a species-specific behavior; rhesus monkey TRIM5α protects against HIV but not against their natural simian immunodeficiency virus (SIV) and, vice versa, human TRIM5α is efficient against the animal retroviruses MLV and EIAV and partially inhibits HIV-2, but not HIV-1 (Hatziioannou et al., 2004; Perron et al., 2007). The “specificity” of restriction is entirely determined by the C-terminal PRY-SPRY domain of the protein and, particularly, by its amino acid 332. For instance, a hybrid isoform of the human TRIM5α with the PRY-SPRY domain from the rhesus monkey protein or with mutations R332G/R335G rendered it highly active against HIV-1 and have been widely used in LV transduction experiments to demonstrate protection against HIV-1 *in vitro* and *in vivo* (Anderson and Akkina, 2008; Pham et al., 2010; Jung et al., 2015). In 2018, Dufour et al. performed R332G/R335G mutagenesis of endogenous TRIM5α in HEK 293T cells with CRISPR/Cas9 and a ssODN repair matrix and obtained 5.6% of cells bearing both mutations in one allele but no cells with biallelic KI (Dufour et al., 2018). Later on, the same group used Cas9 RNP and ssODN electroporation to isolate one Jurkat cell clone with biallelic R332G/R335G mutation and several clones with a monoallelic mutation in TRIM5α. In contrast to monoallelic KI, cells with biallelic mutations were markedly protected from HIV-1 infection, an effect that was further enhanced by IFNβ treatment (about 40-fold for cells with biallelic KI). Importantly, LV-delivered TRIM5α (R332G/R335G) did not respond to IFN stimulation and inhibited HIV replication in Jurkat cells more than one order of magnitude less efficiently compared with biallelic KI coupled with IFN stimulation (Désaulniers et al., 2020).

In summary, although the application of these strategies to the treatment of people living with HIV is still in the distant future and requires KI optimization, these experiments demonstrate a clear advantage of editing endogenous genes whose expression is physiologically regulated compared with therapeutic genes, such as modified restriction factors (see above) or C34-CXCR4 (Leslie et al., 2016), that have been cloned in LV for constitutive overexpression.

### Knock-In of Fusion Inhibitory C-Peptides

C-peptides are short amino acid sequences from the heptad repeats 2 (HR2) of the viral fusion protein gp41 that interfere with the last step of HIV entry (Jiang et al., 1993) by blocking the formation of a six-helix bundle that is required to fuse the viral and cellular membranes (Chen, 2019). A detailed review of the mechanisms of HIV entry blockage using inhibitory proteins and peptides can be found in (Pu et al., 2019). Growing evidence suggests that membrane-anchored gp41 peptides confer dramatically higher levels of protection from HIV infection than soluble analogs. This has been demonstrated both with lipid-modified synthetic peptides (Ingallinella et al., 2009; Ashkenazi et al., 2012; Augusto et al., 2014; Chong et al., 2016; Chong et al., 2017) and peptides encoded genetically (Hildinger et al., 2001; Egelhofer et al., 2004; Liu et al., 2016; Tang et al., 2019). Some genetically encoded C-peptides have entered clinical trials; for instance, C46 alone (Van Lunzen et al., 2007) in Germany or in combination with shRNA specific to CCR5 (sh5) (Delville et al., 2019) in France, or synthetic C34-PEG_4_-Chol in the UK (Quinn et al., 2017). While C-peptides’ high effectiveness against HIV has been proven in different animal models (Burke et al., 2015; Peterson et al., 2016), no encouraging data from patient trials have yet been reported. In the above-mentioned studies, the peptides have been delivered with lenti- or retroviral vectors in the context of sufficient large GPI-proteins such as the low-affinity nerve growth factor receptor (LNGFR) or decay-accelerating factor (DAF), and no data on HDR-based HIV inhibitory peptide delivery have been published so far.

Earlier, we described a novel method of gene-edited cell selection called SORTS (Surface Oligopeptide knock-in for Rapid Target Selection), which is based on the targeted knock-in of epitope tags embedded in CD52 (Zotova et al., 2019b). CD52 is the shortest human GPI-anchored protein that can effectively deliver peptides to the plasma membrane, where they serve as markers for the fluorescence-activated cell sorting (FACS) isolation of edited cells. SORTS has been successfully applied to select the biallelic KO of genes encoding intracellular or secreted proteins when antibody-negative sorting of live cells was impossible. In this study, we also designed the KI of the hemagglutinin (HA) tag into HIV-1 capsid protein p24 and used the SORTS procedure to demonstrate the isolation of CEM and primary CD4^+^ T cells with effectively inactivated HIV-1 proviruses. Unlike previous studies that relied entirely on indel formation, the targeted integration of the short polyA termination signal from the β-globin gene designed in the transgenic construct downstream of the selection marker created both a high level of surface HA expression and a robust silencing of proviral DNA.

We next hypothesized that providing the selected cells with a protective peptide would render “cured” cells resistant to repeat infection with HIV and adapted SORTS for the delivery of fusion inhibitor peptides (Maslennikova et al., 2022). Three of the seven C-peptides cloned in the CD52 molecule, MT-C34, 2P23, and HP23L, displayed the strongest cell protection from HIV. These peptide constructs, in conjunction with the generated peptide-specific antibodies (Abs), were used to build a CRISPR/Cas9 HDR-based platform for the generation and selection of lymphocytes with peptides integrated into the *CXCR4* gene. Using model CEM/R5 lymphoid cells, we demonstrated different options for target gene modification, such as the *in-frame* fusion of peptides with the N-terminus of CXCR4, their separate and fully functional expression, CXCR4 KO, and the expression of two peptides with synergistic effects. All of these strategies proved viable and fully protected CEM/R5 cells from both X4- and R5-tropic strains of HIV-1 and even from gp41 mutants. Nevertheless, the first attempts to reproduce these results in primary lymphocytes were unsuccessful due to low HDR levels. However, after donor DNA optimization (extension of homology arms, switching from PCR products to plasmids, and the addition of protospacer and SV40 DNA transport sequences) and Cas9 modification through the addition of a nuclear localization signal (NLS), the levels of HDR were improved by up to 35% in CEM/R5 cells and 4–5% in primary CD4^+^ lymphocytes. Comparative analysis of MT-C34 peptide expression *via* KI and LV transduction revealed that while LV offered more effective and less toxic peptide delivery to the primary lymphocytes, KI resulted in a higher level of MT-C34 expression and stronger protection from HIV infection.

In summary, SORTS-based gp41 peptide KI is advantageous because it provides a high level of peptide expression *via* premature transcriptional termination; likely reduced immunogenicity due to inducible expression from the CXCR4 or LTR promoter; combined, strong protection mediated by concomitant coreceptor or provirus inactivation; and a low probability of escape mutant emergence as it is not based on indel formation. Notably, many of the listed benefits are not attributed to LV. Further study is needed to achieve clinically relevant HDR levels and to minimize off-targeting.

### Knock-In-Based Reprogramming of B-Lymphocytes to Produce Anti-HIV bNAbs

B lymphocytes, immune cells that produce antibodies, are classified into three major subsets: follicular B cells, marginal zone (MZ) B cells, and B1 cells. The follicular B cells elicit the T-helper-dependent response and supply the serum with most of its antigen-specific antibodies. At the first extrafollicular stage, after BCR engagement and MHC-II restricted antigen presentation to CD4^+^ T cells, B cells receive activation signals, proliferate, and differentiate into short-lived plasma cells (SLPCs), producing mainly low-affinity IgM, albeit class-switch recombination (CSR) may occur at this stage. The next follicular stage is characterized by the migration of SLPCs into the follicles of the lymph nodes and spleen and their active proliferation with the formation of germinal centers (GCs). In GCs, the Ig genes of plasmablasts undergo CSR and somatic hypermutations (SHM) that result in the secretion of high-affinity IgG, IgA, or IgE. Ultimately, SLPCs differentiate into long-lived plasma cells (LLPCs) and memory B cells, which later migrate to the bone marrow where they persist and secrete protective antibodies (Kometani and Kurosaki, 2015; Nutt et al., 2015).

HIV infection in a small subset of HIV-infected people, called HIV elite controllers, is controlled by the production of broadly neutralizing antibodies (bNAb) after many years of anti-Env antibody tuning and maturation mediated by SHM. However, many years of vaccine development have failed to induce potent bNAbs in animal models or human trials (reviewed in (Pollara et al., 2017)). In two closely related studies published by teams from the US (Hartweger et al., 2019) and Israel (Nahmad et al., 2020), the possibility of engineering mature B lymphocytes with CRISPR/Cas9 to produce recombinant anti-HIV bNAbs has been elegantly demonstrated. To this end, the authors designed a transcription unit encoding the full light chain and a variable segment of the heavy chain of a human bNAb, separated by the ribosome skipping sequence P2A. To reconstitute the Ig heavy chain, the 3’-end of the transgene was flanked by a splice donor (SD) enabling its joining to one of the constant heavy-chain regions of the target gene. The expression of recombinant bNAbs was driven either by an endogenous promoter, for which the coding sequence of the transgene was placed *in frame* with an upstream exon and flanked by the 5’ splice acceptor (SA) site, or by an external Ig promoter placed in the transgene with an upstream SA stop cassette or polyA terminator to prevent VDJ transcription. The designed cassette was knocked-in in the J-C intron located between the VDJ and C segments of the *IgH* gene, turning off the expression of the target gene. In addition, to avoid mispairing the transgenic heavy chain with endogenous kappa light chains, KI was coupled to *IgK* KO. This design was tested on mature primary mouse and human B lymphocytes with KI efficiency ranging from 0.1% to 9%. Strikingly, an adaptive transfer of a few thousand correctly-edited mouse B cells to syngeneic mice followed by prime-boost immunization with a cognate antigen elicited the production of bNAbs at concentrations exceeding those detected in HIV elite controllers. More importantly, transgenic B cells have been shown to migrate, proliferate, and differentiate in GCs; undergo CSR, as different isotypes and classes of bNAb are detected in sera; and undergo SHM, producing bNAbs with affinity-modulating mutations. This example confirms that low-efficient HDR can be sufficient to elicit therapeutically relevant effects.

Unlike the genetic strategies intended to confer HIV resistance exclusively to edited cells, transgenic B cells protect all virus permissive cells *via* the secretion of bNAbs. The idea of expanding the protection against HIV-1 from edited to bystander cells *via* the secretion or shedding of short C-peptides (Egerer et al., 2011; Maslennikova et al., 2022) or sCD4 (Falkenhagen et al., 2017) has also been explored. However, we (Maslennikova et al., 2022) and others have failed to achieve a reasonable level of protection due to problems related to the inefficient entry of a short peptide into the secretory pathway, which can partially be circumvented with peptide concatamerization (Egerer et al., 2011). Thus, engineering B cells with CRISPR/Cas9 to produce bNAbs is a very promising technology and it would be interesting to see its effectiveness in protecting primates from HIV.

### HDR Approaches to Engineer Anti-HIV CAR T Cells

Chimeric antigen receptor (CAR) T cells have been widely applied for cancer treatment, with the most success in treating B-cell lymphomas. CAR generally contains an antigen-specific ectodomain and an activating intradomain consisting of a signaling CD3zeta-chain from TCR and one or two cytoplasmic domains from costimulatory molecules such as CD28 and 4-1BB. T cells redirected for universal cytokine-mediated killing (TRUCK), CAR T cells of the last generation, additionally encode IL-12, which recruits NK cells, macrophages, and other innate immune cells, enhancing the anti-tumor response and elimination of cancer cells. The advances in CAR T therapy for HIV, its limitations, and side effects have been discussed in a recent review by Jinxin Qi et al. (Qi et al., 2020).

Currently, two molecules, CD4 and its derivatives or anti-HIV single-chain bNAbs, are used to construct CAR. This leaves anti-HIV CAR T cells vulnerable to HIV infection. To overcome this obstacle, Hale et al. used megaTAL to knock in bNAb-based CAR into the *CCR5* gene of primary CD3^+^ T cells (not separated from CD4^+^ cells). These cells showed significantly higher levels of HIV suppression than CAR T cells engineered *via* LV transduction (Hale et al., 2017). The CRISPR/Cas9 system is widely applied to knock out the TCR receptor or checkpoint molecules (PD-1) during CAR cell preparation, alleviating the graft-versus-host reaction and enhancing the anti-tumor effect, respectively. TCR-redirected T lymphocytes engineered with CRISPR/Cas9 and infused into patients with refractory cancers exhibited durable engraftment with low cytotoxicity or signs of cytokine release syndrome (Stadtmauer et al., 2020). The development of similar CRISPR/Cas strategies to cure HIV can be expected; however, no such studies have been reported. Future work in this field will need to solve the problems of CAR cell infection, viral escape from recognition by CAR or TCR receptors, the insensitivity to cells infected with latent HIV, and the low HDR efficiency using large CAR constructs.

## Fighting HIV With CRISPR/Cas9-Based Gene Regulation

Deactivated Cas9 (dCas9), lacking nuclease activity, is a tool for the DNA-specific delivery of different protein modules that can alter the transcription of the target gene either through epigenetic DNA modification or by changing the recruitment of transcription factors. These modifications are rarely stable and are not inherited by daughter cells. When applied to HIV treatment, they can be divided into virus activation strategies for shock and kill therapy, viral suppression, and restriction factor activation.

### Shock and Kill

“Shock and kill” refers to an approach in which activating latent HIV leads to the killing of cells hosting the virus through either virus-induced suicidal death or immune attack, while ongoing HAART protects intact cells from infection *de novo* (Deeks, 2012). In 2018, Ben Berhout’s lab published a comprehensive review of HIV latency and shock therapy using latency-reversing agents (LRAs) or genetic methods (Darcis et al., 2018). A potent reactivation of latent HIV reservoirs has been demonstrated by applying dCas9 fused to VP64, a tetramer of the Vp16 transactivation domain from the herpes simplex virus (Limsirichai et al., 2016; Saayman et al., 2016); a SAM complex that additionally recruits the p65 subunit of NF-κB and the human heat shock factor 1 (HSF-1) activating domain through the MS2 aptamer (Zhang et al., 2015; Bialek et al., 2016; Limsirichai et al., 2016); or SanTag, in which dCas9 is fused to multiple copies of the GCN4 peptide that are recognized by the anti-GCNA single-chain Ab linked to VP64 (Bialek et al., 2016; Ji et al., 2016). Typically, sgRNAs targeting the U3 region of 5’-LTR about 100 to 200 bp upstream of the transcription start site (TSS), which corresponds to the NF-kB binding site, display the highest levels of viral activation. Applying two sgRNAs (Zhang et al., 2015; Bialek et al., 2016) or a combination of CRISPR activators (CRISPRa) with LRAs (Limsirichai et al., 2016) resulted in the synergistic amplification of HIV expression. All of these studies consistently demonstrated that the SAM complex outperforms the other modules in LTR activation. Notably, Zhou et al. described a new SPH system combining the SunTag and SAM complexes that activated many genes 2- to 3-fold more efficiently than previous activators (Zhou et al., 2018). It would be interesting to test this system’s utility for HIV shock and kill. Unlike transcription factor recruiters, dCas9 fused to the p300 human acetyltransferase catalytic core provides an alternative method for gene activation *via* H3 histone acetylation at position K47, triggering epigenetic chromatin remodeling. Limsirichai et al. showed that when dCas9-p300 is targeted to the U3 modulatory region, it activates HIV expression better than the SAM complex (Limsirichai et al., 2016). Because epigenetic changes will lead to sustained HIV reactivation, the therapeutic effect of histone acetylation can be expected to be greater than that from the transient expression of activator modules.

In summary, the dCas9-mediated activation of latent HIV is a potent strategy to reduce the size of the viral reservoir, is safer than wtCas9 as it does not introduce changes in the DNA sequence, and is specific compared with LRAs. However, difficulties of large activation complex delivery (Saayman et al., 2016), the transient and incomplete nature of activation, and the non-responsiveness of some integrated proviruses impede its clinical utility. A combination of CRISPRa with recently reported T4 bacteriophage-based nanoparticles carrying recombinant CD4DARPin (Batra et al., 2021) that specifically and potently activate CD4+ T cells may improve shock and kill therapy.

### Suppression of HIV Transcription

The repression of HIV transcription by CRISPRi (or “block-and-lock” therapy) presents an alternative to other anti-HIV gene-targeting approaches. It can be achieved either by binding dCas9 to the target gene to sterically interfere with transcription initiation or elongation or by using a special repressive domain that will remodel chromatin structure for more efficient and prolonged gene silencing. In 2016, Di Qu et al. used dCas9 to map the regions of HIV 5’-LTR suitable for suppression and found the SP1 core promoter to be the most appropriate (Qu et al., 2016). In 2020, Alex Olson et al. applied dCas9 coupled with a transcriptional repressor domain from the Krüppel associated box (KRAB) for HIV silencing. They selected two sgRNAs targeting the 5’-LTR at positions 397 and 518, which flank TSS, as the most potent repressors of HIV transcription and latent HIV-1 provirus reactivation. The degree of HIV repression was correlated to the increased level of methylated histone H3K9me3 and decreased level of acetylated histone H3 (Olson et al., 2020). Recently, Nader Alerasool et al. reported a new KRAB repressor element from the ZIM3 protein that outperformed many currently existing CRISPRi severalfold (Alerasool et al., 2020), however, it has not yet been tested for HIV silencing.

### Activation of HIV Restriction Factors

The cytidine deaminases APOBEC3G (A3G) and APOBEC3B (A3B) are cellular proteins that restrict HIV replication by converting dC to dU at the minus strand of viral DNA during reverse transcription, resulting in the G-to-A hypermutation of nascent viral DNA (Okada and Iwatani, 2016). While HIV accessory protein Vif efficiently binds to and degrades A3G in proteasomes, A3B is resistant to Vif and, therefore, active against wild type (wt) HIV. However, unlike A3G, A3B in human primary lymphocytes is neither expressed nor induced by type II IFNs or other stimuli (Refsland et al., 2010). Bogerd et al. designed sgRNAs specific for A3G or A3B promoters and showed significant activation of both restriction factors with dCas9-SAM, especially when two sgRNAs per gene were applied (Bogerd et al., 2015). As expected, the induced A3G and A3B proteins blocked infection with ΔVif HIV-1, but only A3B activation inhibited the replication of wt HIV-1 and is, therefore, therapeutically relevant. Another protein, BST-2, acts as a restriction factor for many viruses by tethering budding virions to the surface of infected cells. HIV-1 has evolved the Vpu protein, which counteracts BST-2. Zhang et al. demonstrated the efficient activation of BST-2 followed by dCas9-SAM/sgRNA lentiviral delivery to non-lymphoid and lymphoid cell lines. This inhibited wt and even ΔVpu HIV-1 virion production (albeit less efficiently for the mutant virus) (Zhang et al., 2019).

Thus, boosting the expression of endogenous restriction factors is an attractive strategy for fighting HIV, although the rationale for its use is uncertain. For example, A3B was overexpressed in a wide spectrum of human cancers, where it can sustain mutational processes and cancer development (Zou et al., 2017). Likewise, BST-2 upregulation was related to enhanced cancer cell migration, invasion, proliferation, and drug resistance (Mahauad-Fernandez and Okeoma, 2016). Moreover, we (Zotova et al., 2019a) and others (Jolly et al., 2010) demonstrated that by tethering mature virions on the cell surface, BST-2 can promote cell-to-cell transmission of HIV-1.

## Perspectives in CRISPR/Cas Anti-HIV therapy and Concluding Remarks

In addition to the most popular NHEJ- and HDR-based applications of spCas9 and its catalytically deactivated protein chimeras described above, many newer editing technologies have been demonstrated to be efficient against HIV. Knipping et al. recently reported the inactivation of HIV coreceptors with base editors. Using cytosine base editors, they introduced premature stop codons in the *CCR5* and *CXCR4* genes and mutated the *CCR5* start codon using adenine base editors. The efficiency of edits detected in primary CD4^+^ T cells and HSPCs was high, around 90%, albeit biallelic KO was lower (Knipping et al., 2022). Unlike NHEJ-mediated gene KO, base editing produces much fewer off-target mutations because of the lack of dsDNA breaks, offering precise DNA modification as in HDR-related editing, and, therefore, can be considered a promising technology. We have already discussed the advantages of using Cas12a for HIV proviral DNA excision with a single sgRNA over Cas9. Liu et al. successfully applied Cas12a to mutate CCR5 as well and render cells resistant to HIV and found fewer off-target mutations produced by Cas12a compared with using Cas9 (Liu et al., 2020). Because Cas12a and, especially, its orthologs Cas12j and Cas12f are much smaller than Cas9, their improved delivery with size-restricted vehicles, such as AAV, viral, and nonviral particles (Briolay et al., 2021), is highly probable and should motivate their development and application in the future. As alternatives to the genetic and epigenetic silencing of HIV, two recent reports demonstrated the efficient suppression of HIV replication and reactivation at the mRNA/gRNA level with Cas13a (Yin et al., 2020) and Cas13d (Nguyen et al., 2021). Interestingly, Cas13a degraded gRNA packed into viral particles. Although this ability can be applied to fight other RNA viruses, obviously it is not suitable for HIV eradication.

**Figure 2:**
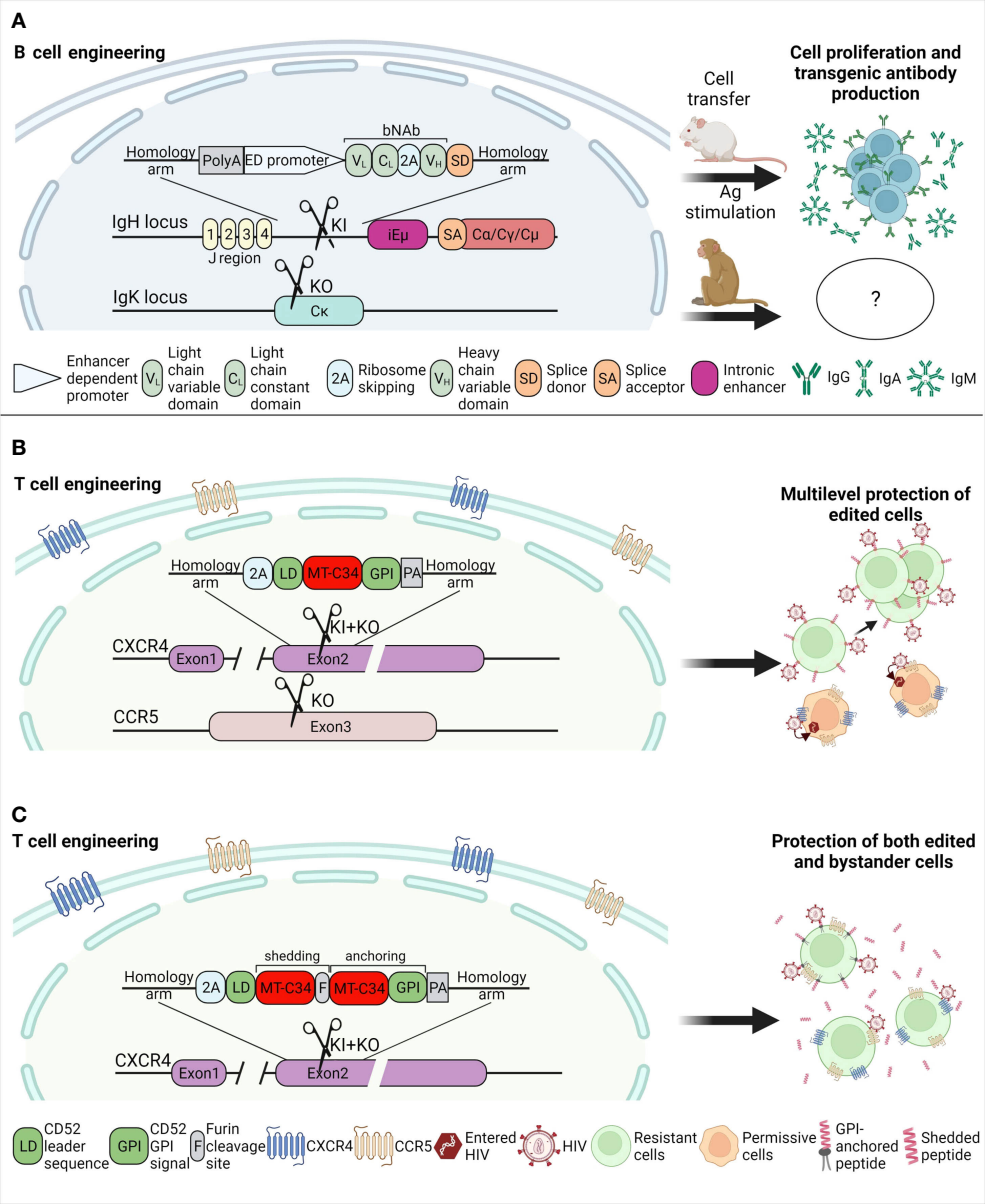
Perspective HDR-based strategies that provide strong and broad protection against HIV. **(A)** Mature B lymphocytes engineered *via* knock-in of bNAb into *IgH* locus are capable to proliferate after adoptive transfer to syngeneic mice and immunization with cognate antigen and produce a high titer of anti-HIV Ig of different classes. Hopefully, preclinical trials on primates and clinical studies will be successful as well. The concept of full-length light chain and variable domain heavy chain knock-in-out at *IgH* locus was adapted from the papers (Hartweger et al., 2019; Nahmad et al., 2020). The single-chain antibody was introduced between VDJ and C segments that preserves mechanism of Ig class switch, while additional *IgK* gene KO prevents mispairing of light chain with transgenic heavy chain. **(B)**. Knock-in-out of MT-C34 peptide from gp41 protein into the *CXCR4* locus with a parallel ablation of *CCR5* gene ensures full tropism-independent protection of engineered CD4^+^ T cells from HIV. **(C)**. A combination of secreted and GPI-anchored forms of MT-C34 *via* peptide concatemeric construct knocked-in into *CXCR4* gene will provide the selection of CD4^+^ T cells that are resistant to HIV and secrete the MT-C34 peptide which will shield non-edited cells from HIV infection. The GPI-anchored C-peptide KI concept was described previously in our paper (Maslennikova et al., 2022) and here it was complemented with CCR5 KO and peptide secretion strategy which was reported earlier and called SAVE (Egerer et al., 2011). The symbol and pictogram labels (grouped for panels **B, C**) are shown at the bottom of pictures. All images were created with BioRender.

When selecting HIV targets, the inhibition of entry, particularly through coreceptor ablation, expression of fusion inhibitor peptides, sCD4, or bNAbs, results in the strongest cell resistance to HIV, while shRNA knockdown or restriction factor expression usually display modest antiviral effects. While individual coreceptor KO confers resistance to tropism-specific HIV, fusion peptides protect against a broad range of viral strains. Their small size provides higher levels of KI than bNAb or CAR. On the other hand, engineering B cells with bNAbs is promising because it does not require high levels of HDR due to the amplification of the therapeutic effect by prime-boost immunization (**Figure 2A**). The clear advantage of this technique is that monoclonal Ab protects all permissive cells in the organism, while the resistance mediated by GPI-peptides or coreceptor KO is conferred exclusively to edited cells. The modification of a transgene for the production of both GPI-anchored and secreted peptides through concatamerization and furin-mediated peptide cleavage might circumvent this limitation (**Figures 2B, C**). The monoclonal nature of secreted Ab, as well as the usage of peptides from viral sequences, raise concerns about the emergence of resistant HIV. To avoid this possible adverse outcome, B cells could be engineered with two or three bNAbs and peptide mimetics such as 2P23 could be used. The other way to circumvent resistant HIV is to provide multilayer protection; for instance, by combining inhibitory peptide expression with CXCR4 and CCR5 KO. The strategies that target viral sequences, such as HIV DNA excision, may encounter problems with HIV diversity and the selection of effective sgRNAs (Yoder, 2019). In such cases, a personalized approach in sgRNA design would help make therapy more effective (Dampier et al., 2018; Chung et al., 2021).

Finally, problems intimately related to programmable nuclease technology, off-target effects, chromosomal rearrangements, and low levels of HDR in primary cells should be resolved in the future to make gene-editing therapies in humans safer and more efficient. Recent advances in the field of CRISPR/Cas RNP delivery using different viral nanoparticle systems such as Nanoblades (Mangeot et al., 2019; Gutierrez-Guerrero et al., 2021), NanoMEDIC (Gee et al., 2020), HIV Gag-Cas9 cleavable fusion (Hamilton et al., 2021), gesicles (Campbell et al., 2019) or Vpr-mediated nuclease encapsidation (Indikova and Indik, 2020) look very promising as provide high efficiency, low toxicity, and reduced off-targeting during editing of clinically relevant types of primary cells. Additionally, pseudotyping viral nanoparticles enables delivery of CRISPR/Cas complexes to specific cell types as was shown by using HIV Env for delivery of β-2 microglobulin gene KO into CD4 lymphocytes (Hamilton et al., 2021). Thus, enveloped particles can open up a new avenue for direct *in vivo* use of CRISPR/Cas technologies and, particularly, for delivery genetic resistance specifically to HIV sensitive cells.

## Author Contributions

Both authors listed, have made substantial, direct and intellectual contribution to the work, and approved it for publication.

## Funding

This work was financially supported by the grant 075-15-2019-1661 from the Ministry of Science and Higher Education of the Russian Federation.

## Conflict of Interest

The authors declare that the research was conducted in the absence of any commercial or financial relationships that could be construed as a potential conflict of interest.

## Publisher’s Note

All claims expressed in this article are solely those of the authors and do not necessarily represent those of their affiliated organizations, or those of the publisher, the editors and the reviewers. Any product that may be evaluated in this article, or claim that may be made by its manufacturer, is not guaranteed or endorsed by the publisher.
